# Impact of the COVID-19 Pandemic on Adolescent Self-Harm: Based on a National Emergency Department Information System

**DOI:** 10.3390/ijerph20054666

**Published:** 2023-03-06

**Authors:** Ju-Hyeon Park, Young-Woo Seo, Seungbum Chae

**Affiliations:** 1 Department of Emergency Medicine, School of Medicine, Daegu Catholic University, Daegu 42472, Republic of Korea; 2 Department of Orthopedic Surgery, School of Medicine, Daegu Catholic University, Daegu 42472, Republic of Korea

**Keywords:** adolescents, COVID-19, self-harm, suicide

## Abstract

Republic of Korea’s suicide rate is the highest among Organization for Economic Co-operation and Development countries. In Republic of Korea, suicide is the leading cause of death among young people aged 10–19 years. This study aimed to identify changes in patients aged 10–19 years who visited the emergency department in Republic of Korea after inflicting self-harm over the past five years and to compare the situations before and after the outbreak of the COVID-19 pandemic. Analysis of government data revealed that the average daily visits per 100,000 were 6.25, 8.18, 13.26, 15.31, and 15.71 from 2016 to 2020, respectively. The study formed four groups for further analysis, with the population divided by sex and age (10–14 and 15–19 years old). The late-teenage female group showed the sharpest increase and was the only group that continued to increase. A comparison of the figures 10 months before and after the outbreak of the pandemic revealed a statistically significant increase in self-harm attempts by only the late-teenage female group. Meanwhile, visits (per day) in the male group did not increase, but the rates of death and ICU admission increased. Additional studies and preparations that account for age and sex are warranted.

## 1. Introduction

Adolescent suicide is a major global public health issue [1]. In 2020, approximately 1,500,000 adolescents and young adults (ages 10–24 years) died worldwide, and suicide was the second leading cause of death [2]. In Republic of Korea, the suicide rate was 24.6 per 100,000 in 2020, the highest among OECD member countries [3]. Indeed, suicide is the fifth leading cause of death in Korea [4]. By age, suicide is the most common cause of death among people in their teens [4].

Since the outbreak of the COVID-19 pandemic, concerns about social isolation, economic crises, and fears of infectious diseases have deepened [5,6]. Adolescents—who are not only mentally and physically immature compared with adults, but who also live a life confined to the home and school—particularly faced a greater risk of social isolation. Studies on adolescents and young adults have reported that depression, anxiety, and suicidal thoughts have increased since the beginning of the pandemic [5,6,7]. Despite such risks, a review article found no evidence that self-harm increased after the outbreak of the pandemic [8]. Among Korean adolescents, suicide continued to increase from 2016 to 2019, particularly among mid-adolescents (14–16 years old) compared with late-adolescents (17–19 years old) [9]. A study using trend prediction analysis reported that the number of patients under the age of 34 years increased more than the predicted value after the start of the pandemic [10]. Another study, based on the Korea Youth Risk Behavior Web-based Survey, reported that compared with 2019, the odds ratio of self-harm attempts decreased in 2020, after the start of the pandemic, to 0.64 [95% CI, 0.58–0.70] [11].

The conflicting research results may be attributed to differences in data investigation methods or subjects. In general, the first place a patient visits after attempting self-harm is an emergency department [12]. As such, many studies on self-harm have been conducted based on emergency department data [9,13,14,15,16,17,18,19,20,21]. After the start of the COVID-19 pandemic, studies on self-harm and suicide in adolescents by age and sex based on national emergency department data are still insufficient. Therefore, we compared and analyzed trends before and after the outbreak of the COVID-19 pandemic by using data from the National Emergency Department Information System (NEDIS).

## 2. Methods

### 2.1. Data Collection and Target Population

From the NEDIS data, collected and managed by the National Emergency Medical Center in Korea, we analyzed data from 2016 to 2020 on patients aged 10–19 years who visited emergency rooms in Korea after inflicting self-harm. The first case of COVID-19 in Korea was reported on 19 January 2020; subsequent cases occurred sporadically thereafter, before increasing in number rapidly after the 31st COVID-19 patient was detected on 18 February 2020 [22]. From this point, the nationwide response against the outbreak of COVID-19 began in Korea, and consequently, rapid social changes began in parallel [22]. Therefore, we decided to use the data after March 2020 as the data after the outbreak of the COVID-19 pandemic.

### 2.2. Study Methods

We collected the following: patients’ age, sex, visit date, visit time, region, method of self-harm attempt (one choice), transportation, Korean Triage and Acuity Scale (KTAS) score, hospitalization, inpatient ward, emergency department (ED) exit time, discharge date, discharge time, and treatment results. By age, the patients were grouped into 10–14 and 15–19 years, in accordance with the regulations of the National Emergency Medical Center in Korea. Visit dates were classified by season, and regions were divided into metropolitan and non-metropolitan. As for transportation to visit the ED, we divided it into 119 ambulance, other ambulance, other vehicle, on foot, and unknown. The KTAS is scored by health care professionals on a scale of 1–5 depending on severity, by comprehensively evaluating the main symptom, injury mechanism, pain level, general condition, and vital signs. The lower the score, the more severe the condition. In accordance with the emergency and non-emergency classification method of the Ministry of Health and Welfare [23], we classified scores 1 to 3 as “emergency” and scores 4 to 5 as “non-emergency.” Methods of self-harm attempt were classified into ingestion, cutting/stabbing, drowning, asphyxia/hanging, fall, and others. Final outcome was classified into survival, death, and unknown. Based on the collected data, we analyzed the number of ED visits per 100,000 population according to the population by year, age group, sex, and region provided by Statistics Korea, Korea’s central government organization for statistics. This study was approved by the Institutional Review Board of Daegu Catholic University Hospital (IRB number: CR-22-035-L). Informed consent was not required.

### 2.3. Statistics

We generated a summary of the population characteristics using descriptive analysis. We obtained the values of mean and standard deviation (SD) for quantitative variables, and the values of frequency and percent for qualitative variables. For the normality test, we used the Shapiro–Wilk test for continuous variables. To compare patients who visited the emergency room after self-harming by year, we performed a one-way ANOVA or Friedman test according to whether the normality was satisfied for quantitative variables, and we performed the chi-squared test for qualitative variables. For the comparison before and after the start of the COVID-19 pandemic according to sex and age, we conducted a two sample *t*-test or Mann–Whitney U test for quantitative variables depending on whether normality was satisfied, and a chi-squared test for qualitative variables. All *p*-values less than 0.05 were considered statistically significant. All tests were performed as a two-tailed test. We performed statistical analyses using IBM SPSS Statistics for Windows, version 21.0 (SPSS Inc., Chicago, IL, USA).

## 3. Results

### 3.1. Five-Year Comparison (2016–2020)

From 1 January 2016 to 31 December 2020, a total of 18,037 teenage patients visited the ED owing to self-harm or suicide. Table 1 shows the data provided by Statistics Korea sorted by year, region, age, and sex during the five years. The mean (±SD) of daily visits per 100,000 population indicated statistically significant differences, and was increasing every year. Based on the patient’s age group (i.e., 10–14 and 15–19 years) and sex (i.e., male and female), four groups were created and analyzed: early teenage male (ETM), late teenage male (LTM), early teenage female (ETF), and late teenage female (LTF). In the case of LTF, the results were statistically significantly different. Only this group showed continuously increasing mean daily visits.

By visit season, summer showed the highest rate of ED visits in all years between 2016 and 2020, except in 2019. Regarding the ratio between metropolitan and non-metropolitan, the number of patient visits was smaller in metropolitan than in non-metropolitan regions. Regarding transportation for ED visits, “other vehicle” accounted for the most frequent type, followed by the public 119 ambulance. The proportion of patients with a KTAS score of 1–3, indicating an emergency, increased every year from 2016 to 2020. As for self-harm methods, cutting/stabbing and ingestion accounted for the majority of cases.

Although we found no statistical difference in the comparison of the ratio of treatment outcomes, the number of dead patients increased every year (except for 2017). Table 1 also lists the hospitalization rate and rate of admission to the intensive care unit (ICU) among inpatients, as well as the mean length of stay in the ED and mean admission days.

### 3.2. Comparison before and after the COVID-19 Outbreak

For the comparison before and after the outbreak of the COVID-19 pandemic, we excluded the data from 2016 to 2018, which showed a drastic increase in adolescent self-harm and suicide. We used data from March to December 2019 as a control group and the data from March to December 2020 (i.e., after the outbreak of the COVID-19 pandemic on 19 February 2020) as the study group. In our analysis, we divided the cases into four groups according to sex and age group.

As shown in Table 2, in the ETM, LTM, and ETF, the number of patients who visited the ED for self-harm attempts decreased after the COVID-19 pandemic, and only the LTM showed no statistical difference. However, the mean ED visits per day in the LTF increased by 1.32 visits per day from before the pandemic to after, showing a statistical difference. By season, mean ED visits per day in the LTF increased in summer and autumn, showing statistically significant differences.

By residence, the mean ED visits per day in the LTF in metropolitan regions increased, whereas that in the ETM and ETF in non-metropolitan regions decreased, showing a statistically significant difference. In addition, in the comparison of mean visits per day by region per group, only the LTF in metropolitan regions showed a statistically significant increase.

By transportation type for ED visits, the use of the 119 ambulance increased after the COVID-19 pandemic in all groups except the LTF, showing statistically significant differences. The LTM group showed the highest rate of ED visits using the 119 ambulance. In the comparison according to KTAS scores, we found that after the outbreak of the COVID-19 pandemic, the ratio of emergency cases (1–3 points) for the LTM and ETF groups increased by 11.5% and 6.5%, respectively. In addition, the ratio of emergency cases (1–3 points) was 4.2% to 17.7% higher for females than for males.

The most common methods of self-harm attempt were ingestion and cutting/stabbing in females, in that order; whereas the reverse order was reported in males. In all four groups, the total ratio of the combination of ingestion and cutting/stabbing decreased after the start of the pandemic, and all groups except for the LTM showed statistically significant differences in this ratio. In particular, the ETM increased from 8.8% to 17.8%, whereas the ETF increased from 6.0% to 11.7%. In the comparison of treatment outcomes, we found statistical differences for only the ETM; mortality in the ETM more than doubled. Among inpatients, the rate of admission to the ICU increased in the ETM and LTM. However, female groups did not show differences before and after the pandemic outbreak. Regarding the ED stay time and length of hospital admission/stay days, we found no statistically significant differences in all four groups. Meanwhile, males’ ED stay times were 52 to 129 min shorter compared with females.

## 4. Discussion

Korea has the highest suicide rate in the world, and suicide is the number one leading cause of death among teenagers. For comparison before and after the start of the COVID-19 pandemic, this study investigated the trend of change over a sufficient period of time before the pandemic; based on the results, the comparison periods before and after the pandemic were determined. To obtain accurate and meaningful results, we intended to reflect the demographic changes in Korea. For example, the country’s fertility rate has fallen over the past decades, and the combined total fertility rate became 0.84 in 2020, which decreased by 0.08 compared with 2019 [24]. Therefore, we performed statistical analyses based on data per 100,000 population (median value of the year) by year, region, sex, and age provided by Statistics Korea. To the best of our knowledge, this study is the first to compare adolescent self-harm before and after the start of the COVID-19 pandemic through statistical analyses of NEDIS data from the National Emergency Medical Center and demographic changes in Korea.

From 2016 to 2019, the number of patients per day who visited the ED for self-harm drastically increased—to 2.45 times—coinciding with that reported in another study, 2.36 times [9], performed in Korea at the same time. Another study reported a higher increase, of 5.54 times [13]. The former research [9] was conducted using national data, as was the current study, whereas the latter study [13] analyzed single-center data. Such a large variation might be attributed to the use of a single center design. In 2020, when COVID-19 began to spread worldwide, the number of ED visits per day was 15.71 (±6.777) in Korea, and the steep increasing trend declined.

Based on sex, the total number of ED visits per day by teenage males increased by 65.8% over the four years from 2016 to 2019. During the same period, the number of ED visits per day by teenage females increased about threefold, and was consistent with previous reports [9,25]. Regarding such results, Lee et al. [9] argued that depression-related symptoms increased only in females after 2005 in Korea, that female adolescents are more sensitive to the socioeconomic status of their parents than their male counterparts, and that female adolescents feel more stress over their physical and psychological changes during adolescence. As implied by our results, although the increase in ED visits slowed in 2020, public officials or researchers in charge of suicide prevention and mental health should pay attention and be cautious of the steady rise in teenagers’ self-harm over the years, particularly among female adolescents.

By season, the suicide rate in autumn of 2019 was the highest at 27.9%, whereas in all other years summer had the highest rate. According to a study on the seasonal effect of suicide in Korea from 1991 to 2015, most died by suicide in spring, and then gradually decreased throughout the seasons, being the lowest in winter [26]. However, such a tendency has gradually decreased as the years have passed [26]. In our study, from 2016 to 2020, when we only focused on the season of spring, 2017 was the year with the highest number of self-harm attempts. However, our results from the study years (i.e., 2016 to 2020) were inconsistent with the trend noted above. In many other countries, studies have noted differences in seasonal trends based on age and sex, with variations depending on the time and country of the study [27,28,29,30,31,32,33,34,35,36,37,38,39,40,41,42,43,44]. Further research is necessary to determine whether the difference between the current and prior studies is due to a change in the seasonal trend of suicide attempts in Korea or differences according to age groups.

Methods of attempting self-harm vary by country [45]. In Korea [13,15,20,46], ingestion and cutting/stabbing account for the majority. In our study, these two methods accounted for the majority as well. The rates of drowning and asphyxia/hanging, equivalent to 2 points on the risk rescue rating scale (RRRS) [47], were 2.9% and 3.6%, respectively, whereas that of falls, scoring 3 points on the RRRS, ranged from 3.7% to 4.9%. Falls were the only method that showed consistent as well as slightly declining percentages, but the number of ED visits owing to falls per 100,000 population steadily increased from 2016 to 2018.

Since 2016, the number of teens visiting the ED for self-harm has consistently increased. Therefore, to make a more accurate comparison before and after the start of the COVID-19 pandemic, we classified March to December 2020 as the post-pandemic period, and March to December 2019 as the pre-outbreak period. We then further divided the cases into four groups according to sex (male and female) and age (10–14 and 15–19 years), and then conducted a comparative analysis.

After the COVID-19 outbreak, as shown in Table 2, the mean ED visits per day in the ETM and ETF decreased by 50% and 23%, respectively; while those in the LTM decreased slightly from 3.65 (±2.863) to 3.64 (±3.252), with this decrease being statistically insignificant (*p*-value = 0.065). The mean in the LTF increased by 14.1%; the LTF was the only group to show an increase after the outbreak of the COVID-19 pandemic. Such a result is similarly reported in systematic review articles: more countries have had no change or a decrease in self-harm or suicide rates in the months after the start of the COVID-19 pandemic [8,48]. By season, mean visits per day of the two groups in the early teens (i.e., ETM and ETF) showed a decrease in spring and summer, whereas no statistical difference was found between the two late teen groups (i.e., LTM and LTF) in the same season. Considering that Korea’s first outbreak of the COVID-19 pandemic occurred in late winter to early spring, our finding of a decrease in self-harm in the period immediately after the outbreak matched that found in previous studies [49,50,51]. Additional follow-up studies are needed to investigate whether such seasonal variation is due to the pandemic of an infectious disease or to the weakening seasonal variability of self-harm patients in Korea [26].

In the comparison of self-harm attempt methods, ingestion was the most frequently used method by females both before and after the start of the pandemic, whereas cutting/stabbing was the most frequently used method by males. Compared with female, the ratio of RRRS 2 points was 1.7% to 9.5% higher in males, and that of RRRS 3 points was 0.6% to 3.4% higher in males. After the start of the pandemic, such differences tended to increase, and only the ETF showed a steep increase in RRRS 3 points from 3.9% to 9.3%. Males tend to try more aggressive self-harm methods than females. After the start of the pandemic, such a tendency has become more prominent. In the mortality comparison, the ETM increased from 5.4% to 13.6%, and the LTM increased from 2.0% to 3.1%, whereas the female groups showed a slight decrease. Only the ETM showed a statistically significant difference. In addition, the rate of admission to the ICU among inpatients increased from 21.9% to 48.1% in the ETM, and from 28.5% to 41.7% in the LTM. Considering self-harm methods, prognosis, and ICU admission rate, in the same period, the KTAS score for an “emergency” for males (i.e., 1–3 points) was 4.2% to 14% lower than that for females. Such a result may be attributed to the tendency among females to complain more about symptoms than males. In addition, the same difference may influence the longer ED stay in females than males in the same group by approximately 1 to 2 h.

This study has a few limitations. The first is the limitation of the data provided. The patients’ socioeconomic status, motivation for self-harm, detailed treatment process, detailed method of self-harm, and interview details were not available. Given the computerized nature of collecting ED data across Korea, there was a high possibility of missed data. Thus, analyses on the causes of self-harm attempts were not possible. Moreover, additional analysis was not possible because age information was only provided in terms of age group. The second limitation is that patients who did not directly harm themselves but who visited the ED or outpatient clinic for fear, depression, or anxiety were not included in this study. A study investigating such categories is also needed.

Despite these limitations, our study is valuable for being the first to identify trends in the years before the COVID-19 pandemic, reflect the population structure, and examine the impact of the COVID-19 pandemic on teenage self-harm based on national-level data in Korea. A notable finding was that the LTF showed the steepest, and a continuing, increase in the number of self-harm patients, and such an increase was maintained even after the start of the pandemic, consistent with a previous systematic review [48]. However, we were not able to reveal whether such results were due to the influence of the pandemic or to the continuity of other factors that have affected the increase in self-harm in the LTF group. Government institutions in Korea, such as the Suicide Prevention Center and the Ministry of Health and Welfare, as well as the clinical departments of psychiatry, emergency medicine, and trauma care, should be aware of these changes and prepare for them. Meanwhile, among male teenage patients, while the number of self-harm patients did not increase after the pandemic, the use of more aggressive methods, the increase in ICU admission rate, and the poor prognosis should also be considered and prepared for. Further studies are recommended to identify the causes of these results, in order to prevent self-harm and prepare long-term countermeasures.

## 5. Conclusions

In Korea, self-harm among teenagers has increased rapidly over the past few years, with self-harm among females in their late teens (15–19 years old) having increased most rapidly. In addition, self-harm among females only in their late teens increased, in comparison, before and after the start of the COVID-19 pandemic. In contrast, self-harm among males did not increase before and after, but the ICU admission rate and the mortality rate both increased. Consequently, follow-up studies are required, particularly on the causes of, prevention of, and preparation for these findings.

## Figures and Tables

**Table 1:** Comparison of patients who visited an emergency department for self-harm in Korea from 2016 to 2020.

	2016 (N = 2287)	2017 (N = 2986)	2018 (N = 4839)	2019 (N = 5587)	2020 (N = 5752)	*p*-Value
Total † (per day)	6.25 ± 3.883	8.18 ± 4.321	13.26 ± 6.804	15.31 ± 6.445	15.71 ± 6.777	<0.001 *
Male † (per day)	2.63 ± 2.279	3.30 ± 2.696	3.88 ± 3.158	4.36 ± 3.178	3.93 ± 3.243	<0.001 *
Female † (per day)	3.61 ± 3.108	4.88 ± 3.329	9.37 ± 5.907	10.95 ± 5.253	11.78 ± 5.879	<0.001 *
Early-teen male † (per day)	0.33 ± 0.965	0.52 ± 1.071	0.59 ± 1.336	0.69 ± 1.376	0.39 ± 0.955	<0.001 *
Late-teen male † (per day)	2.30 ± 2.132	2.78 ± 2.477	3.30 ± 2.764	3.67 ± 2.772	3.54 ± 3.119	<0.001 *
Early-teen female † (per day)	0.55 ± 1.417	0.61 ± 1.247	2.06 ± 1.247	2.01 ± 2.451	1.56 ± 2.027	<0.001 *
Late-teen female † (per day)	3.06 ± 2.589	4.28 ± 3.039	7.32 ± 4.498	8.94 ± 4.692	10.22 ± 5.460	<0.001 *
Season						<0.001 *
Spring	589 (25.7)	799 (26.7)	1057 (21.8)	1383 (24.8)	1230 (21.4)	<0.001 *
Summer	631 (27.6)	844 (28.3)	1517 (31.4)	1500 (26.9)	1651 (28.7)	<0.001 *
Autumn	630 (27.6)	727 (24.4)	1325 (27.4)	1558 (27.9)	1634 (28.4)	<0.001 *
Winter	437 (19.1)	616 (20.6)	941 (19.4)	1145 (20.5)	1236 (21.5)	<0.001 *
Region						0.019 *
Metropolitan	1005 (44.0)	1307 (43.8)	2111 (43.6)	2327 (41.6)	2574 (44.7)	
Non-Metropolitan	1281 (56.0)	1680 (56.2)	2727 (56.4)	3261 (58.4)	3179 (55.3)	
Transportation						<0.001 *
119 ambulance	907 (39.7)	1179 (39.5)	1897 (39.2)	2260 (40.5)	2485 (43.2)	
Other ambulance	138 (6.0)	168 (5.6)	238 (4.9)	195 (3.5)	171 (3.0)	
Other vehicle	1226 (53.6)	1633 (54.7)	2686 (55.5)	3100 (55.5)	3065 (53.3)	
On foot	11 (0.5)	7 (0.2)	10 (0.2)	22 (0.4)	29 (0.5)	
Unknown	4 (0.2)	0 (0.0)	8 (0.2)	8 (0.1)	3 (0.1)	
KTAS						<0.001 *
1–3	1252 (54.8)	1642 (55.0)	2713 (56.1)	3166 (56.7)	3432 (59.7)	
4–5	1033 (45.2)	1344 (45.0)	2127 (43.9)	2421 (43.3)	2316 (40.3)	
Method						<0.001 *
Ingestion	902 (39.4)	1128 (37.8)	2100 (43.4)	2417 (43.3)	2439 (42.4)	
Cutting/stabbing	1109 (48.4)	1478 (49.5)	2172 (44.9)	2637 (47.2)	2699 (46.9)	
Drowning	27 (1.2)	25 (0.8)	36 (0.7)	49 (0.9)	60 (1.0)	
Asphyxia/Hanging	55 (2.4)	73 (2.4)	110 (2.3)	111 (2.0)	126 (2.2)	
Fall	112 (4.9)	131 (4.4)	207 (4.3)	206 (3.7)	263 (4.6)	
Others	84 (3.7)	151 (5.1)	215 (4.4)	164 (2.9)	165 (2.9)	
Outcome						0.227
Survival	2215 (96.9)	2923 (97.9)	4717 (97.5)	5455 (97.7)	5605 (97.4)	
Death	59 (2.6)	43 (1.9)	89 (1.8)	98 (1.8)	107 (1.9)	
Unknown	13 (0.6)	20 (0.7)	33 (0.7)	33 (0.6)	40 (0.7)	
Admission (among total)	638 (27.9)	754 (25.2)	1173 (24.2)	1344 (24.1)	1390 (24.2)	<0.001 *
To ICU (among admission)	223 (35.0)	250 (33.2)	320 (27.3)	460 (34.2)	565 (40.7)	<0.001 *
Mean ER stay time (h:mm)	4:30 ± 6:16	4:22 ± 5:44	4:50 ± 6:01	4:47 ± 5:57	4:40 ± 5:57	0.011 *
Mean Admission Days	10.1 ± 15.20	9.3 ± 14.89	9.6 ± 14.95	9.4 ± 15.15	10.0 ± 14.52	0.778

KTAS: Korean Triage and Acuity Scale. ICU: Intensive Care Unit. *p*-values were obtained by one-way ANOVA or chi-squared test. Values in parentheses represent percent. *: Statistically significant at *p* < 0.05. †: Values presented as mean frequency ± standard deviation per 100,000.

**Table 2:** Comparison before and after the start of the COVID-19 pandemic by sex and age.

		Male (N = 2617)			Female (N = 7258)		
		Before N = 1386)	After (N = 1231)	*p*-Value	Before (N = 3501)	After (N = 3757)	*p*-Value
ET †	Per day	0.78 ± 1.465	0.39 ± 0.949	<0.001 *	2.10 ± 2.500	1.61 ± 2.096	0.009 *
LT †	Per day	3.75 ± 2.863	3.64 ± 3.252	0.065	9.34 ± 4.779	10.66 ± 5.579	0.002 *
Season (per day)							
ET †	Spring	0.77 ± 1.531	0.21 ± 0.540	0.001 *	2.27 ± 2.679	1.25 ± 1.940	0.003 *
	Summer	1.06 ± 1.579	0.53 ± 1.084	0.009 *	1.94 ± 2.529	1.29 ± 1.652	0.042 *
	Autumn	0.75 ± 1.429	0.52 ± 1.202	0.244	2.13 ± 2.276	2.28 ± 2.431	0.672
	Winter	0.07 ± 0.270	0.10 ± 0.292	0.641	1.97 ± 2.577	1.68 ± 2.273	0.645
LT †	Spring	3.51 ± 3.148	3.55 ± 3.004	0.937	8.48 ± 4.819	8.36 ± 4.700	0.871
	Summer	3.77 ± 2.782	4.38 ± 3.108	0.168	9.53 ± 4.857	11.75 ± 5.159	0.003 *
	Autumn	4.05 ± 2.852	3.28 ± 3.662	0.115	10.20 ± 4.207	11.88 ± 5.748	0.026 *
	Winter	3.50 ± 2.209	2.75 ± 2.783	0.250	8.84 ± 5.679	10.71 ± 6.724	0.241
Region							
ET	Metropolitan	92 (38.5)	61 (51.7)	0.018 *	280 (43.5)	207 (41.9)	0.579
	Non-metropolitan	147 (61.5)	57 (48.3)		363 (56.5)	287 (58.1)	
LT	Metropolitan	465 (40.5)	466 (41.9)	0.521	1207 (42.2)	1501 (46.0)	0.003 *
	Non-metropolitan	682 (59.5)	647 (58.1)		1651 (57.8)	1763 (54.0)	
Region (per day)							
ET †	Metropolitan	0.30 ± 0.844	0.20 ± 0.636	0.100	0.91 ± 1.506	0.68 ± 1.171	0.029 *
	Non-metropolitan	0.48 ± 1.213	0.19 ± 0.676	<0.001 *	1.18 ± 1.995	0.94 ± 1.616	0.092
LT †	Metropolitan	1.52 ± 1.505	1.52 ± 1.624	0.992	3.95 ± 2.551	4.90 ± 3.398	<0.001 *
	Non-metropolitan	2.23 ± 2.417	2.11 ± 2.610	0.577	5.40 ± 4.041	5.76 ± 4.380	0.286
Transportation							
ET	119 ambulance	61 (25.5)	46 (39.0)	0.007 *	202 (31.4)	208 (42.0)	0.001 *
	Other ambulance	18 (7.5)	0 (0.0)		17 (2.6)	18 (3.6)	
	Other vehicle	160 (66.9)	72 (61.0)		423 (65.8)	268 (54.1)	
	On foot	0 (0.0)	0 (0.0)		1 (0.2)	1 (0.2)	
	Unknown	0 (0.0)	0 (0.0)		0 (0.0)	0 (0.0)	
LT	119 ambulance	529 (46.2)	593 (53.3)	0.001 *	1167 (40.8)	1297 (39.7)	0.186
	Other ambulance	43 (3.8)	30 (2.7)		100 (3.5)	91 (2.8)	
	Other vehicle	570 (49.7)	489 (44.0)		1569 (54.9)	1851 (56.7)	
	On foot	4 (0.3)	0 (0.0)		16 (0.6)	21 (0.6)	
	Unknown	0 (0.0)	0 (0.0)		7 (0.2)	3 (0.1)	
KTAS							
ET	1–3	111 (46.4)	59 (49.6)	0.576	367 (57.1)	314 (63.6)	0.027 *
	4–5	128 (53.6)	60 (50.4)		276 (42.9)	180 (36.4)	
LT	1–3	523 (45.6)	636 (57.1)	<0.001 *	1810 (63.3)	1999 (61.3)	0.073
	4–5	624 (54.4)	477 (42.9)		1049 (36.7)	1262 (38.7)	
Method							
ET	Ingestion	61 (25.5)	36 (30.5)	0.003 *	309 (48.2)	210 (42.5)	0.008 *
	Cutting/stabbing	143 (59.8)	58 (49.2)		274 (42.7)	209 (42.3)	
	Drowning	3 (1.3)	0 (0.0)		3 (0.5)	2 (0.4)	
	Asphyxia/Hanging	6 (2.5)	14 (11.9)		10 (1.6)	10 (2.0)	
	Fall	12 (5.0)	7 (5.9)		25 (3.9)	46 (9.3)	
	Others	14 (5.9)	3 (2.5)		20 (3.1)	17 (3.4)	
LT	Ingestion	357 (31.1)	368 (33.1)	0.053	1423 (49.8)	1500 (46.0)	0.037 *
	Cutting/stabbing	660 (57.5)	582 (52.3)		1194 (41.8)	1479 (45.3)	
	Drowning	19 (1.7)	18 (1.6)		23 (0.8)	33 (1.0)	
	Asphyxia/Hanging	30 (2.6)	42 (3.8)		47 (1.6)	41 (1.3)	
	Fall	45 (3.9)	67 (6.0)		95 (3.3)	118 (3.6)	
	Others	36 (3.1)	36 (3.2)		77 (2.7)	92 (2.8)	
Outcome							
ET	Survival	223 (93.3)	102 (86.4)	0.016 *	626 (97.4)	480 (97.4)	0.873
	Death	13 (5.4)	16 (13.6)		12 (1.9)	8 (1.6)	
	Unknown	3 (1.3)	0 (0.0)		5 (0.8)	5 (1.0)	
LT	Survival	1114 (97.1)	1075 (96.6)	0.087	2817 (98.5)	3203 (98.1)	0.081
	Death	23 (2.0)	34 (3.1)		33 (1.2)	37 (1.1)	
	Unknown	10 (0.9)	4 (0.4)		9 (0.3)	24 (0.7)	
Admission							
ET	Admission (among total)	64 (26.8)	27 (22.9)	0.016 *	158 (24.6)	141 (28.5)	0.568
	Admission to ICU (among admission)	14 (21.9)	13 (48.1)	0.012	45 (28.5)	37 (26.2)	0.665
	ET stay time (h:mm)	2:53 ± 4:25	3:48 ± 6:04	0.138	5:02 ± 5:28	5:21 ± 6:41	0.477
	Admission day	9.5 ± 12.45	7.5 ± 8.62	0.506	9.8 ± 12.62	12.6 ± 14.76	0.139
LT	Admission (among total)	253 (22.1)	264 (23.7)	0.759	701 (24.5)	784 (24.0)	0.188
	Admission to ICU (among admission)	72 (28.5)	110 (41.7)	0.002 *	287 (40.9)	333 (42.5)	0.052
	ED stay time (h:mm)	3:57 ± 5:33	4:10 ± 5:09	0.361	5:02 ± 6:10	5:02 ± 5:53	0.960
	Admission day	8.9 ± 14.99	10.0 ± 14.22	0.468	9.0 ± 14.93	10.0 ± 15.51	0.254

ET: early teen (10–14 years old), LT: late teen. KTAS: Korean Triage and Acuity Scale. ICU: Intensive Care Unit. *p*-values were obtained by *t*-test or chi-squared test. Values in parentheses represent percent. *: Statistically significant at *p* < 0.05. †: Values presented by mean frequency ± standard deviation per 100,000.

## Data Availability

Third party data. Data were obtained from the National Emergency Medical Center and are available at https://www.e-gen.or.kr/nemc/main.do (accessed on 4 January 2023) with the permission of the National Emergency Medical Center. Data can be obtained by submitting the documents required by the National Emergency Medical Center, including a research plan and IRB approval-related documents, undergoing the review process, and then paying a fee.
